# An Outer Membrane Vesicle-Adjuvanted Oral Vaccine Protects Against Lethal, Oral *Salmonella* Infection

**DOI:** 10.3390/pathogens10050616

**Published:** 2021-05-18

**Authors:** Jaikin E. Harrell, Jonathan R. Kurtz, David L. Bauer, J. Timothy Prior, Patrick S. Gellings, Lisa A. Morici, James B. McLachlan

**Affiliations:** Department of Microbiology and Immunology, Tulane University School of Medicine, New Orleans, LA 70112, USA; jharrel3@tulane.edu (J.E.H.); jkurtz1@tulane.edu (J.R.K.); dbauer3@tulane.edu (D.L.B.); jprior2@tulane.edu (J.T.P.); pgellings@tulane.edu (P.S.G.); lmorici@tulane.edu (L.A.M.)

**Keywords:** *Salmonella*, mucosal immunity, outer membrane vesicles, vaccine

## Abstract

Non-typhoidal salmonellosis, caused by *Salmonella enterica* serovar Typhimurium is a common fecal-oral disease characterized by mild gastrointestinal distress resulting in diarrhea, chills, fever, abdominal cramps, head and body aches, nausea, and vomiting. Increasing incidences of antibiotic resistant invasive non-typhoidal *Salmonella* infections makes this a global threat requiring novel treatment strategies including next-generation vaccines. The goal of the current study was to formulate a novel vaccine platform against *Salmonella* infection that could be delivered orally. To accomplish this, we created a *Salmonella*-specific vaccine adjuvanted with *Burkholderia pseudomallei* outer membrane vesicles (OMVs). We show that adding OMVs to a heat-killed oral *Salmonella* vaccine (HKST + OMVs) protects against a lethal, oral challenge with *Salmonella*. Further, we show that opsonizing anti-*Salmonella* antibodies are induced in response to immunization and that CD4 T cells and B cells can be induced when OMVs are used as the oral adjuvant. This study represents a novel oral vaccine approach to combatting the increasing problem of invasive *Salmonella* infections.

## 1. Introduction

Vaccines are one of the most successful public health initiatives in reducing morbidity and mortality of infectious diseases and have led to the eradication of diseases like smallpox and polio [1]. While some vaccines are comprised of live, attenuated viruses or bacteria, others may be formulated using inactivated whole organisms or subunit components. Inactivated and subunit vaccines are often poorly immunogenic and require inclusion of an adjuvant to boost the magnitude and breadth of the adaptive immune response [2]. The most common adjuvant currently in use are aluminum salts (alum) that have been in use for nearly a century. While alum can drive a robust antibody response, it does not induce cellular immunity or generate an immune response at mucosal surfaces [3]. Stimulating a mucosal immune response is critical for next-generation vaccines, as most pathogens are first encountered at the mucosal surface [4]. One way to drive mucosal immunity is to deliver vaccines directly into the mucosal tissue where immunity is most desired. These mucosal adjuvant delivery systems are capable of producing both a systemic and mucosal response [5]. While some mucosal adjuvants excel at driving pulmonary mucosal immunity when delivered intranasally or intratracheally, few can induce similar immunity when delivered orally, likely due to the tolerogenic mechanisms at play in the gut [6]. As such, novel mucosal adjuvants should be explored to understand their role in inducing greater protection against mucosal pathogens, particularly those that infect via the oral route.

One possible mucosal adjuvant is outer membrane vesicles (OMVs), which are naturally produced by Gram-negative bacteria and contain a variety of pathogen-associated molecular patterns, giving them an intrinsic adjuvant effect [7]. In nature, OMVs are used by bacteria for a variety of purposes, including biofilm formation, cargo delivery, communication, and nutrient acquisition [8]. These naturally occurring OMVs can be isolated and purified and used in vaccines as adjuvants. For example, OMVs derived from *Neisseria meningitidis* are a major component of the FDA-approved vaccine Bexsero [9]. OMVs from other species, such as *Burkholderia pseudomallei*, have been used as a vaccine platform against infection with various *Burkholderia* species and have been shown to produce both humoral and cell-mediated immune responses [10,11,12]. *B. pseudomallei* OMVs have also been shown to be safe and effective in mice and rhesus macaques [13] and have recently been shown to function as a potent adjuvant [14]. Unlike alum, OMVs, like those from *Vibrio cholerae*, are able to be given directly at a mucosal surface and induce long-term local and systemic immune responses [15]. Here, we sought to use *B. pseudomallei*-derived OMVs to adjuvant an orally delivered *Salmonella enterica* serovar Typhimurium (hereafter *S*. Typhimurium) vaccine in order to enhance protection against a lethal, oral *Salmonella* infection.

*S*. Typhimurium is a fecal-oral pathogen that is the causative agent of non-typhoidal salmonellosis that is typically a self-limiting infection; however, in certain parts of the world, *S*. Typhimurium can become invasive. This invasive infection, or invasive non-typhoidal salmonellosis (iNTS), is characterized by bacteremia, fever, hepatosplenomegaly, and respiratory symptoms with a lack of enterocolitis [16]. While some effective vaccines against *S*. Typhi, the causative agent of typhoid fever, exist, there are currently no licensed vaccines for infection with *S*. Typhimurium. The most effective *S*. Typhi vaccine is a vaccine comprised of tetanus-toxoid conjugated to the Vi capsular antigen of *S*. Typhi. This vaccine provides approximately 80% efficacy and high rates of seroconversion following vaccination [17]. This strategy, while effective for *S*. Typhi, cannot be used for *S*. Typhimurium due to the lack of a capsule. As such, novel vaccine strategies, including mucosally delivered vaccines, should be explored to combat the growing incidence of both non-typhoidal and invasive non-typhoidal salmonellosis.

Parenteral (non-mucosal) administration of most vaccines is not known to illicit robust mucosal immunity, yet it is thought that gut immunity is important for protection against *Salmonella* [18]. Additionally, protective *Salmonella* immunity is classically linked to activation of CD4 T cells [19,20,21], but innate and humoral immunity have also been shown to play a role [22]. In the current study, we exploited the natural route of infection with an OMV-adjuvanted *Salmonella* vaccine to directly target the mucosal sites where *Salmonella* is known to invade [23]. We showed that the addition of *B. pseudomallei* OMVs to an inactivated *S*. Typhimurium vaccine elicits significant protection against a lethal, oral *Salmonella* challenge compared to inactivated vaccine alone. The OMV-adjuvanted vaccine led to increases in antigen-specific T and B cells and induced anti-*Salmonella* antibodies that were more adept at inducing opsonophagocytosis of *Salmonella* by macrophages compared to non-adjuvanted groups. These findings suggest the *B. pseudomallei* OMVs can successfully be used as an oral adjuvant to elicit protection and to induce cellular and humoral immunity to *Salmonella* and may offer a novel adjuvant approach to elicit protective immunity at what is normally considered a tolerogenic immunization route.

## 2. Results

### 2.1. OMV-Adjuvanted Inactivated S. Typhimurium Vaccine Protects Against Lethal, Oral Challenge with S. Typhimurium

**Figure 1:**
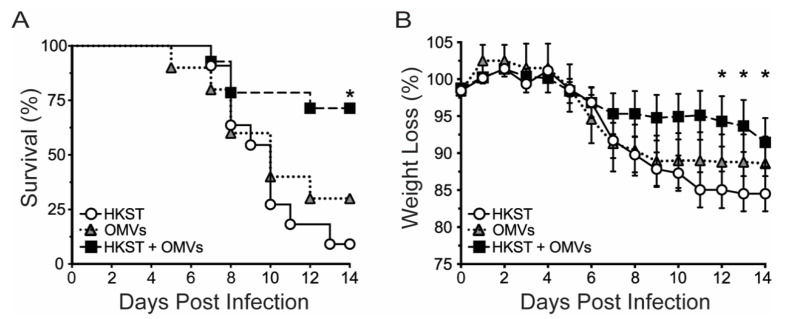
Addition of *B. pseudomallei* OMVs to heat-killed *S*. Typhimurium protects against death and weight loss following a lethal, oral challenge of *S*. Typhimurium. C57Bl/6 mice were orally immunized and boosted with 3 × 10^8^ CFU of heat-killed *S*. Typhimurium and/or 10 µg of Bp82 OMVs. Fourteen days post boost, mice were orally infected with 1 × 10^6^ CFU of *S*. Typhimurium. (**A**) Kaplan–Meier survival following infection. (**B**) Post infection, mice were monitored for weight loss. Data are represented as mean ± SEM, *, *p* < 0.05; Statistical analyses were performed using either a Mantel–Cox test (**A**) or a two-way ANOVA using a Tukey’s test for multiple comparisons (**B**). Results shown are a combination of two experiments, N = 10–14.

Wild-type C57Bl/6 mice are highly susceptible to oral infection with *S*. Typhimurium due to a G169D substitution in the natural resistance-associated macrophage protein 1 (NRAMP-1) gene (also Slc11a1). This non-functional NRAMP-1 cannot control intracellular *Salmonella,* resulting in bacterial replication and fatal infection. We hypothesized that oral immunization of these mice with the heat-killed (inactivated) *S*. Typhimurium vaccine (HKST) adjuvanted with OMVs would protect mice against infection. To test this, C57Bl/6 mice were immunized in a prime-boost fashion (immunized on day 0, boosted on day 14, and infected on day 28) with 3 × 10^8^ CFU of HKST, 10 µg *B. pseudomallei* OMVs (OMVs) alone, or a combination of HKST and OMVs (HKST + OMVs). Mice were subsequently orally challenged with a lethal dose (1 × 10^6^ CFU) of *S*. Typhimurium and monitored for 14 days for mortality and weight loss as a sign of morbidity. Groups that were immunized with either HKST or OMVs alone showed limited survival over the 14 days; however, the HKST + OMVs group demonstrated significant survival compared to the other groups (Figure 1A). Groups displayed a similar trend for weight loss, with the HKST and OMV only groups losing significantly more weight compared to the HKST + OMVs group (Figure 1B). This data demonstrates that the addition of the OMV adjuvant to heat-killed *S*. Typhimurium vaccine delivered orally resulted in interrelated increased survival and protection from severe weight loss following a lethal, oral challenge.

### 2.2. Oral Immunization with OMV-Adjuvanted Inactivated S. Typhimurium Vaccine Induces Neutralizing Systemic and Mucosal Antibodies

**Figure 2:**
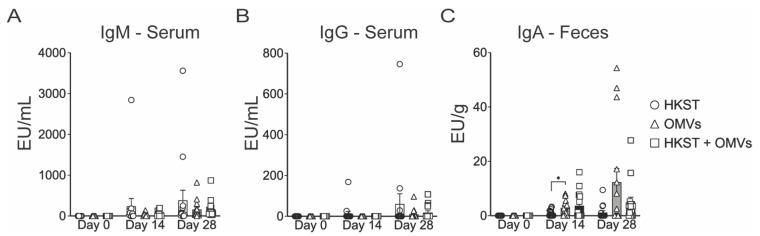
Production of systemic and mucosal *Salmonella*-specific antibodies in both serum and feces following oral immunization. Mice were orally immunized and boosted with 3 × 10^8^ CFU of heat-killed *S*. Typhimurium and/or 10 µg of Bp82 OMVs, and serum and fecal pellets were collected as described in the methods. Plates were coated with whole-cell, heat-inactivated *S*. Typhimurium before the additional of serially diluted serum or fecal homogenate supernatant. (**A**) Serum IgM, (**B**) serum IgG, and (**C**) fecal IgA. For all graphs, data are presented as mean ± SEM; *, *p* < 0.05. Statistical analyses were performed using a two-way ANOVA using a Tukey’s test for multiple comparisons. Results shown are a combination of two independent experiments, N = 15.

We next sought to assess the contribution of the OMVs to the induction of humoral immunity in the oral vaccination model. At each immunization or before infection, blood samples were collected to assess serum IgM and IgG antibodies directed against whole, heat-killed *S*. Typhimurium. IgM was used as a measurement of the initial response to vaccination [24]. By day 28, the HKST + OMVs group produced the highest titers of *S*. Typhimurium-specific IgM (Figure 2A). We next assessed IgG, which indicates that B cells have isotype switched and affinity matured in response to vaccination. Here, the HKST group displayed the highest *S*. Typhimurium-specific IgG response, with the OMV only and HKST + OMVs groups showing minimal production by day 28 (Figure 2B). IgA is also produced following B-cell activation and is mainly found at the mucosal surfaces [24]. Fresh fecal pellets were collected, homogenized, and assessed for IgA as a marker of intestinal secreted IgA, where *S*. Typhimurium is found following oral immunization or infection. On day 14, both OMV only and HKST + OMVs groups had higher levels of IgA, and this trend was maintained on day 28 (Figure 2C). While the OMV group showed the highest IgA response at both day 14 and day 28, these levels did not correspond with survival or protection from weight loss following a lethal, oral challenge (Figure 1).

**Figure 3:**
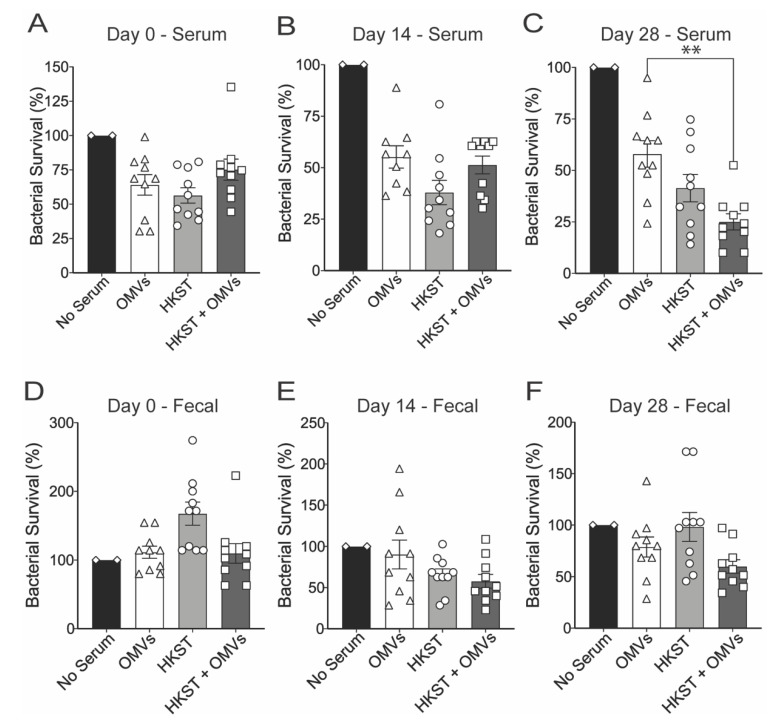
Serum and fecal homogenate supernatant from HKST + OMVs-immunized mice increases bacterial opsonophagocytosis by murine macrophages, leading to bacterial death. Mice were orally immunized and boosted with 3 × 10^8^ CFU of heat-killed *S*. Typhimurium and/or 10 µg of Bp82 OMVs, and serum and fecal pellets were collected as described in the methods. Serum or fecal pellet supernatant was incubated with *S*. Typhimurium and baby rabbit complement. Opsonized bacteria were then incubated with murine macrophages and lysed after 30 min to enumerate surviving bacteria. (**A**–**C**) Percentage of surviving bacteria after opsonophagocytosis with serum collected on day 0 (**A**), day 14 (**B**), and day 28 (**C**). (**D**–**F**) Percentage of surviving bacteria after opsonophagocytosis with fecal pellet homogenate collected on day 0 (**D**), day 14 (**E**), and day 28 (**F**). All data were normalized to no serum or fecal homogenate controls and compared to the adjuvant (OMV only) group. For all graphs, data are presented as mean ± SEM; **, *p* < 0.01. Statistical analyses were performed using a Kruskal–Wallis using a Dunn’s test for multiple comparisons. Results shown are a combination of one experiment, N = 10.

To assess whether there were functional differences in the antibodies, we evaluated the capacity for antibodies to opsonize and induce killing of *Salmonella* using an opsonophagocytic assay. On day 0, the serum from all groups displayed similar killing when normalized to controls without serum (Figure 3A). By day 14, all groups displayed similar values but were slightly lower than those seen on day 0 (Figure 3B). By day 28, however, there was a significant difference between adjuvant alone (OMVs) and the combination of HKST + OMVs, highlighting that serum antibodies produced following immunization with HKST + OMVs promoted increasing killing of *S*. Typhimurium by macrophages (Figure 3C). In addition to serum antibodies, the fecal homogenate, containing IgA *S*. Typhimurium-specific antibodies, was also tested for opsonic activity. On day 0, compared to no fecal homogenate controls, there was an increase in viable bacteria, which could be due to increased uptake of bacteria in the presence of the fecal homogenate (Figure 3D). By day 14, there was a decrease from day 0 values (Figure 3E), and by day 28, the fecal homogenate from HKST + OMV-immunized group promoted the highest percentage of bacterial killing (Figure 3F). While not statistically significant, these data show a similar trend with the survival and protection from weight loss seen in Figure 1.

### 2.3. The Salmonella-Specific CD4 T-Cell Responses Increase in Spleen and MLNs after Oral Immunization

**Figure 4:**
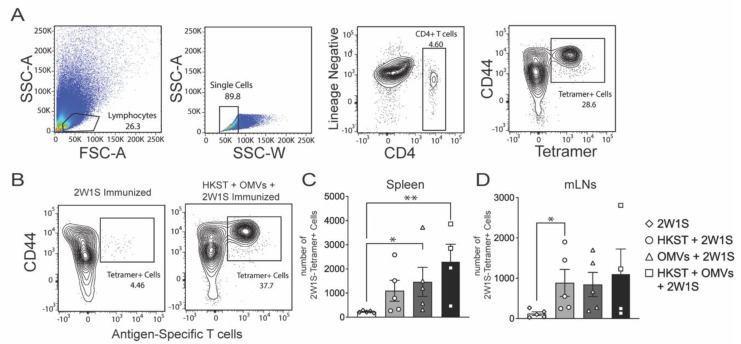
Increase in 2W1S-specific CD4 T cells is seen in spleen and mLNs in response to oral immunization with HKST + OMVs. Mice were orally immunized and boosted with 100 µg of 2W1S peptide alone or combined with 3 × 10^8^ CFU of heat-killed *S*. Typhimurium and/or 10 µg of Bp82 OMVs. (**A**) Lymphocytes were identified by forward or side scatter and only single-cell events were analyzed. Single cells were gated for either CD4 or lineage negative (CD19, CD11b, CD11c, F4/80). The CD4 population was further analyzed for presence of CD44 and the 2W1S tetramer marker. (**B**) Representative flow plots of 2W1S-specific CD4 T cells in the spleen at day 28. (**C**) Total number of 2W1S-specific T cells found in each individual spleen from each mouse. (**D**) Total number of 2W1S-specific T cells found in the mesenteric lymph nodes (mLNs) from each mouse. All groups are compared to the 2W1S-alone control. For all graphs, data are presented as mean ± SEM, *, *p* < 0.05; **, *p* < 0.01. Statistical analysis was performed using a Kruskal-Wallis test with a Dunn’s multiple comparison test. Results shown are representative of two-independent experiments, N = 5.

Following *Salmonella* infection, CD4 T cells are classically identified as the effector cells that expand and control infection [25]. We posited that *Salmonella*-specific CD4 T cells would be most increased in the HKST + OMVs group. To test this, we added a surrogate antigen, called 2W1S, to each vaccine preparation to evaluate the antigen-specific T-cell response induced by immunization (Figure 4A). Previous work has shown that immunization with 2W1S followed by tetramer-enrichment can track changes in a CD4 T-cell population [26,27]. As expected, mice immunized with 2W1S alone showed minimal numbers of 2W1S-specific CD4 T cells in the spleen, as 2W1S alone is not a potent immune stimulator; however, groups immunized with HKST + OMVs + 2W1S displayed a robust 2W1S-specific CD4 T-cell population in the spleen (Figure 4B). In the mesenteric lymph nodes (mLNs), which are the draining lymph nodes for the intestines, groups immunized with either HKST or OMVs only had more 2W1S-specific CD4 T cells than the group immunized 2W1S alone, and the group immunized with HKST + OMVs + 2W1S had slightly more than HKST- and OMV-only groups (Figure 4C). The trend seen in the spleen was similar to that of the mLNs. The group immunized with HKST + OMVs + 2W1S had the highest numbers of 2W1S-specific CD4 T cells (Figure 4D). These results correspond with the increased survival and protection from weight loss seen in Figure 1 and suggest that CD4 T cells could play a role in protection.

### 2.4. Antigen-Specific B Cells Increase and Isotype Switch Following Oral Immunization with the Combination of Heat-Killed Salmonella and B. pseudomallei OMVs

**Figure 5:**
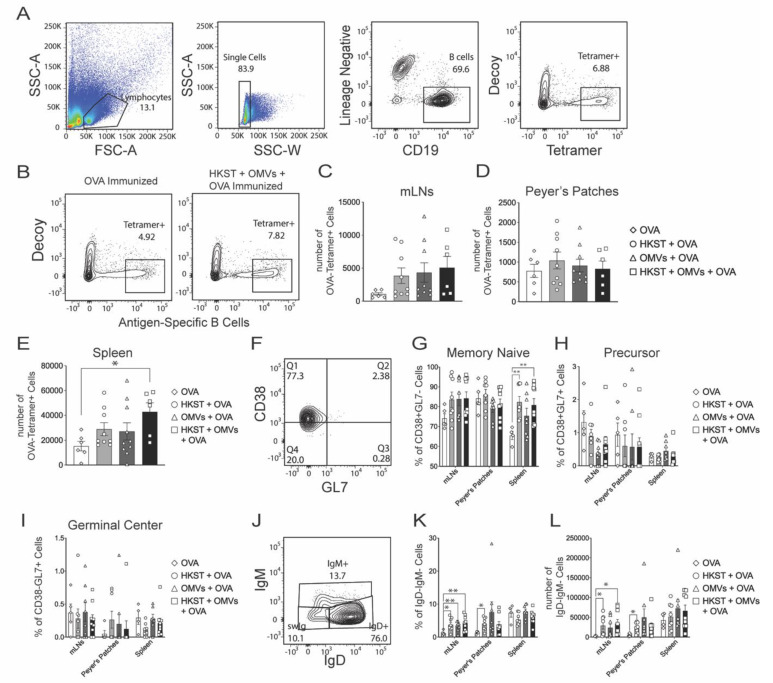
OVA-specific B-cell numbers and isotype switching increase in response to oral immunization. Mice were orally immunized and boosted with 100 µg of OVA protein, 3 × 10^8^ CFU of heat-killed *S*. Typhimurium, and/or 10 µg of Bp82 OMVs, euthanized on day 28, and spleen, mLNs, and Peyer’s patches were harvested and stained with tetramer and decoy. (**A**) Lymphocytes were identified from forward and side scatter and further separated into single-cell events. Single cells were gated to identify B cells (CD19) or other cell populations (CD3, CD11c, F4/80). B cells were analyzed for being specific to the OVA-tetramer or the decoy-tetramer. (**B**) Representative flow plots of OVA-specific B cells in the spleen at day 28. (**C**) Number of OVA-specific B cells in mLNs. (**D**) Number of OVA-specific B cells in Peyer’s patches. (**E**) Number of OVA-specific B cells in spleen. (**F**) Representative flow plot of OVA-specific B cells that express germinal center markers (CD38−, GL7+). (**G**) Percentage of OVA-specific B cells that express the memory naïve (CD38−, GL7−) phenotype. (**H**) Percentage of OVA-specific B cells that express the precursor (CD38+, GL7+) phenotype. (**I**) Percentage of OVA-specific B cells that express the germinal center (CD38−, GL7+) phenotype. (**J**) Gating strategy for isotype-switched, OVA-specific B cells (IgD−, IgM−). (**K**) Percentage of OVA-specific B cells that express the isotype-switched (IgD−, IgM−) phenotype. (**L**) Number of OVA-specific B cells that express the isotype-switched phenotype (IgD−, IgM−). For all graphs, data are presented as mean ± SEM, *, *p* < 0.05; **, *p* < 0.01. Statistical analysis was performed using a one-way ANOVA with Tukey’s multiple comparison test for B-D or a mixed-effect analysis with a Dunnett’s multiple comparison test, with each group compared to OVA alone, for F-H, J-K. Results shown are a combination of two-independent experiments, N = 6-10.

While T cells play an important role in *Salmonella* protection, B cells also play a role in protection independent of antibody production [28]. Here, assessment of antigen-specific B cells was conducted similarly to CD4 T cells; however, in this case, chicken egg ovalbumin (OVA) was the surrogate antigen used to identify antigen-specific B cells with an OVA-specific, B-cell tetramer (Figure 5A). Following the same immunization strategy as above, Peyer’s patches, mLNs, and spleen were harvested and magnetically enriched for OVA-specific B cells. Peyer’s patches were included in addition to the mLNs and spleen due to the high proportion of B cells in this tissue [29]. OVA-specific B cells were detectable both with OVA immunization alone and in combination with adjuvants (Figure 5B). In the mLNs, the HKST + OMVs + OVA group had the highest number of OVA-specific B cells. In addition, the HKST + OVA and OMV + OVA groups displayed more OVA-specific B cells than the OVA group (Figure 5C). This was a similar trend to that seen in the CD4 T-cell population in the mLNs (Figure 4C). In the Peyer’s patches, there were OVA-specific B cells found in each group, but no differences were observed between the groups (Figure 5D). In the spleen, the HKST + OMVs + OVA group also displayed significantly higher numbers of OVA-specific B cells compared to OVA only (Figure 5E).

Following infection or immunization, B cells can form germinal centers in both lymph nodes and secondary lymphoid organs where affinity maturation and isotype switching commonly occur [30]. We next measured the number of OVA-specific B germinal center phenotype cells using the common memory/naïve and germinal center markers CD38 and GL7, respectively (Figure 5F). A majority of cells identified in all of the issues were memory/naïve cells (CD38+,GL7−) with a smaller percentage that adopted the germinal center phenotype (CD38−,GL7+) (Figure 5G–I). Lastly, OVA-specific B cells were assessed for isotype switching, which can occur following antigen stimulation and activation. Naïve B cells express both IgD and IgM on the surface, and those cells that do not express IgD or IgM (IgD−IgM−) are presumed to have class switched and express either IgG, IgA, or IgE (Figure 5J). In the mLNs, all groups had higher percentages of isotype-switched cells compared to the OVA-only group (Figure 5K). In the Peyer’s patches, the HKST group had both a higher percentage and numbers of OVA-specific B cells compared to the group immunized with only OVA (Figure 5K–L). These data suggest that the antigen-specific B cells may contribute to protection, as they agree with the trend seen with survival and protection from weight loss seen in Figure 1.

## 3. Discussion

*Salmonella* Typhimurium infection is typically self-limiting and was estimated to affect 95.1 million people globally in 2017 [31]. In some cases, particularly in malnourished infants and the elderly, this infection can become invasive, cause bacteremia, and have symptoms similar to other febrile illnesses [32,33,34]. This invasive infection is increasing; cases have been rising over the past few decades, with there being 7.5 cases per 100,000 persons compared to 5.9 cases per 100,000 persons in 1990 [31]. Without a vaccine against *S*. Typhimurium, both non-invasive and invasive infections are likely to continue rising and contributing to the global burden of disease. As an oral-fecal pathogen, *S*. Typhimurium is a good candidate for developing a vaccine that directly targets the mucosa. Oral vaccines can be difficult to develop due to the dual challenge of passing through the harsh conditions of the gastrointestinal tract and the potential for inducing tolerance [35]; however, oral vaccines have several advantages over traditionally injected vaccines, including ease of distribution, reduced cost, needle-free administration, and stimulation of mucosal immunity [36]. In the current study, we used heat-killed *S*. Typhimurium adjuvanted with *B. pseudomallei* OMVs to determine if oral immunization could elicit protective mucosal immunity against *Salmonella*. The addition of the OMVs can mimic a live bacterium to stimulate the immune response [14]. Indeed, *B. pseudomallei* OMVs have recently been shown to behave like and elicit similar protection to a live-attenuated vaccine [37]. Here, we hypothesized that the addition of *B. pseudomallei* OMVs to HKST would boost the immune response to *Salmonella* alone and confer protection against challenge. Successful adjuvanting of HKST with *B. pseudomallei* OMVs in an oral vaccine would open the door to explore OMVs as an oral adjuvant and potentially as an adjuvant at other mucosal surfaces.

For these studies, we used WT C57Bl/6 mice, which lack the NRAMP1 gene, making them susceptible to *S*. Typhimurium infection. Following lethal, oral challenge with *Salmonella*, mice that were immunized with the combination of heat-killed *S*. Typhimurium and OMVs had a significant increase in survival compared to either component alone. This highlights that this oral vaccine can elicit protection and that the OMVs can be delivered orally and continue to act as an adjuvant via this route. Oral delivery of a *Salmonella* vaccine is not a new concept—the Ty21a oral vaccine against *S*. Typhi is a live-attenuated vaccine that shows moderate efficacy but only moderate immunogenicity [38]. This highlights the need to identify novel adjuvants for mucosal routes of immunization that can protect against mucosal pathogens.

In addition to showing that an OMV-adjuvanted *Salmonella* vaccine provides protection against challenge, we also explored correlates of protection to offer insight into how this immunization might engage the adaptive immune response. Other groups using *S*. Typhimurium live-attenuated strains or OMVs derived from live-attenuated *Salmonella* have shown protection against challenge and antibody responses but did not explore other potential correlates of protection [39,40,41]. Here, we show that in addition to production of *Salmonella*-specific antibodies, OMV-adjuvanted vaccination elicited antibodies that demonstrate opsonizing functionality. Due to the fact that *S*. Typhimurium can become invasive and lead to bacteremia, production of efficacious systemic antibodies is critical to vaccine success. Taylor and Casadevall highlighted that the importance of bactericidal activity of serum is integral to protection against intracellular pathogens [42,43]. Other groups demonstrated antibody bactericidal activity against non-typhoidal *Salmonella* following immunization or natural infection [44,45]. *Salmonella*-specific antibodies can function to interfere with cellular adhesion, LPS neutralization, antibody-mediated complement lysis, and opsonization [46,47,48,49]. Using an opsonophagocytosis assay to measure killing, we show that serum antibodies produced increased killing following immunization with HKST + OMVs. This increased killing within the macrophages may lead to increased survival during challenge due to protection from bacteremia, which would explain why mice immunized with the HKST + OMVs demonstrated the highest amount of survival.

We were also able to engage the cellular immune response. CD4 T cells are typically identified as the most important effector cells following *Salmonella* infection, and we show that our immunization, which mimics a natural route of infection, induces a vaccine-specific CD4 T-cell response in the spleen, and mLNs that increases when OMVs are included. B cells have also been shown to play an important role in development of protective T-cell responses [28]. We showed that following immunization, we could induce vaccine-specific B cells, and that these B cells were not found in germinal centers. Previous work has shown that following *Salmonella* infection, B cells mature and undergo somatic hypermutation outside of germinal centers, which is consistent with what we showed following immunization [50]. Taken together, this data suggests that the functional antibodies, in addition to T and B cells, are correlates of protection following oral immunization with HKST + OMVs.

This study demonstrates the utility of *B. pseudomallei* OMVs as an oral adjuvant and potentially as an adjuvant at other mucosal surfaces. Administration of OMVs to other mucosal surfaces would be beneficial in defining their adjuvant role and whether they are capable of inducing both humoral and cellular immunity at any site or if this phenomenon is gut specific. In this study, we used *B. pseudomallei* OMVs based on previous literature showing their safety and efficacy both as a vaccine and an adjuvant [13,14,37]. Overall, this study highlights the first time that *B. pseudomallei* OMVs have been employed to adjuvant an oral vaccine. The identification of OMVs as a mucosal adjuvant is critical for two reasons: (1) Alum, which is the most common adjuvant used in parenteral administrations, does not induce mucosal immunity and cannot be given directly at the mucosal surface, and (2) OMVs can adjuvant the response to heat-killed bacteria, offering a potential solution to adjuvanting pathogens that cannot be used as a live-attenuated vaccine.

## 4. Materials and Methods

### 4.1. Ethics Statement

This study was carried out in accordance with recommendations from the Guide for the Care and Use of Laboratory Animals of the National Institutes of Health. All experimental procedures involving animals were approved and performed in compliance with the guidelines established by Tulane University Health Sciences Center’s Institutional Animal Care and Use Committee. Tulane University is accredited by the Association for Assessment and Accreditation of Laboratory Animal Care (AAALAC).

### 4.2. Bacterial Strains

*Salmonella enterica* subsp. *enterica* serovar Typhimurium strain SL1344 (a gift from Marc Jenkins, University of Minnesota) was used for infections as indicated. *Salmonella* was streaked on an LB agar plate, and one clone was selected and grown to late stationary/early log phase for all experiments. *Burkholderia pseudomallei* strain Bp82 is a ΔpurM derivative of *B. pseudomallei* strain 1026b [51].

### 4.3. Mice and Immunizations

C57BL/6 female mice were purchased from Charles River Laboratories (Wilmington, MA, USA) and were maintained under specific-pathogen-free conditions. Animals were fed rodent chow and water ad libitum and allowed to acclimate for 1 week prior to use. Mice (5 to 10 per group) were immunized orally at the age 6 to 8 weeks with 3 × 10^8^ heat-inactivated CFU of *S*. Typhimurium strain SL1344 and/or 10 µg of *B. pseudomallei* OMVs in 200 µL of phosphate buffered saline (PBS) and were boosted 14 days later. For T- and B-cell correlate of protection studies, 100 µg of either 2W1S peptide or ovalbumin (OVA) protein was added to each immunization group. Two weeks following the booster immunization, mice were orally challenged with virulent 1 × 10^6^ CFU of *S*. Typhimurium strain SL1344 in 100 µL of PBS. Mice were monitored for weight loss and signs of morbidity for an additional 14 days following infection. Mice were euthanized if they lost ≥20% of their pre-challenge weight or displayed signs of terminal illness (such as lethargy, being hunched, or lack of grooming). Survival curves were generated, and statistical analyses were performed using a log-rank (Mantel–Cox) test.

### 4.4. Burkholderia pseudomallei Outer Membrane Vesicle Purification

OMVs were purified as previously described with minor modifications [11]. Briefly, *B. pseudomallei* strain Bp82 was freshly streaked from glycerol stock onto *Pseudomonas* isolation agar (PIA) agar and incubated for 48–72 h at 37 °C. A single colony was inoculated into Luria broth (LB) supplemented with 100 µg/mL adenine hydrochloride and 5 µg/mL thiamine hydrochloride (Millipore Sigma, Burlington, MA, USA) and incubated for 16–18 h. The overnight culture was diluted 1:100 in identical media and incubated for 16–18 h at 37 °C until late log phase (OD600 4.5–5). The culture was diluted 1:100 a second time into 500 mL in the same media and cultured in a similar manner. Intact bacteria were pelleted by centrifugation (6000× *g*, 60 min, 4 °C) using an SLA-1500 fixed-angle rotor. Following centrifugation, supernatant was filtered through a 0.22 µm polyethersulfone (PES) to remove remaining bacteria or large fragments. Absence of bacterial contamination was confirmed by incubating supernatant on LB agar for 48–72 h at 37 °C. OMVs in the supernatant were precipitated with 1.5 M ammonium sulfate (Fisher Scientific, Pittsburgh, PA, USA) for 48 h and harvested by centrifugation (15,000× *g*, 90 min, 4 °C) using an SLA-1500 rotor. Crude vesicles were resuspended in 55% sucrose (Millipore Sigma, Burlington, MA, USA) in 10 mM Tris-HCl pH 7.4, layered with a 35–60% sucrose density gradient, and isolated using ultracentrifugation (200,000× *g*, 3 h, 4 °C) using a Fiberlite F50L-8 × 39 fixed-angle rotor. Equal volume fractions were removed from the top, individually subjected to precipitation by TCA and acetone washes, and then evaluated by SDS-PAGE to visualize protein profiles by Coomassie blue staining. Fractions containing identical protein profiles were pooled and subjected to ultracentrifugation (200,000× *g*, 18 h, 4 °C) to obtain highly purified vesicles. Purified vesicles were re-suspended in LPS-free water, visually confirmed by transmission electron microscopy, quantitated by Bradford assay, and stored at −20 °C until use.

### 4.5. Antibody ELISAs

A group of C57BL/6 mice were used for immune correlates (5 mice per group). Mice were bled one day prior to the first immunization (day 1), one day prior to the boost (day 13), and one day prior to challenge (day 27) for the determination of serum antibody responses. Blood was drawn by submandibular vein puncture and collected in BD Microtainer tubes with serum separator additive (BD, Franklin Lakes, NJ, USA). Serum was stored at −80 °C until use. Fresh fecal pellets from mice were collected at the same time as the submandibular vein puncture. Pellets were immediately placed in fecal buffer (1× phosphate buffered saline, Tween-20, 0.5 M EDTA, SIGMAFAST Protease Inhibitor Cocktail Tablet EDTA-Free) on ice. Pellets were homogenized and supernatant was stored at −20 °C until use. Bacteria-specific serum IgM and IgG and bacterial-specific fecal IgA were analyzed by an enzyme-linked immunosorbent assay (ELISA). Ninety-six well microtiter plates were coated with 5 × 10^7^ CFU of heat-inactivated *S*. Typhimurium strain SL1344 per well in coating buffer (0.1 M sodium bicarbonate, 0.2 M sodium carbonate) and were incubated overnight at 4 °C. The plates were washed 3 times with phosphate-buffered saline (PBS) containing 0.05% Tween 20 (PBST). Plates were incubated with 1:100 dilutions of serum or 1:2 dilutions of fecal supernatants overnight at 4 °C. The plates were washed 3 times with PBST and incubated with alkaline-phosphate (AP)-conjugated rat anti-mouse IgG (1:10,000), IgM (1:2000), or IgA (1:2500) for 1 h at room temperature. At the end of incubation, plates were washed 5 times with PBST and developed with TMB 1-Component Microwell Peroxidase Substrate (SeraCare, Milford, MA, USA). After 15–30 min, the reaction was stopped with TMB Stop Solution (SeraCare, Milford, MA, USA) and the absorbance (450 nm) using Epoch2 microplate reader (BioTek, Winooski, VT, USA). The data were analyzed using a sigmoidal, dose-response curve with least-squares fit. The results were quantitated as the number of ELISA units (EU) per milliliter or gram using the average for two sample dilutions closest to the midpoint of the standard curve.

### 4.6. Opsonophagocytosis Assay for Macrophage Survival

RAW 264.7 macrophages (ATCC, Manassas, VA, USA) were routinely grown in complete high glucose Dulbecco’s modified Eagle’s medium (DMEM) (ThermoFisher, Carlsbad, CA, USA) with heat-inactivated fetal bovine serum (Gemini BioProducts, Calabasas, California, USA) and penicillin-streptomycin solution (ATCC, Manassas, VA, USA). Cells were incubated at 37 °C with 5% CO_2_. For infection, 5 × 10^4^ cells per well were plated in a 96-well plate and were left to adhere for 1 h prior to infection. Bacterial inoculum was used at an MOI of 5 (2.5 × 10^5^ CFU) was incubated in the presence or absence of heat-inactivated immune sera from HKST, OMVs, or HKST + OMVs (final concentration of 10%) with baby rabbit complement (Bio-Rad, Hercules, CA, USA) for 30 min at 37 °C with 5% CO_2_. Opsonized bacteria were transferred to plate with cells and incubated for 30 min at 37 °C with 5% CO_2_. After infection, cells were washed, lysed with 0.1% Triton X-100 (MilliporeSigma, Burlington, MA, USA) in PBS, and plated on LB agar for 24 h. Cells were counted after 24 h and values were normalized to unopsonized bacterially infected cells.

### 4.7. Single-Cell Suspension Generation, MHC-II Tetramer Staining, Magnetic Assisted Cell Sorting, Flow Cytometry

Spleens and mesenteric lymph nodes (mLNs) were made into single-cell suspensions by homogenizing organs over 100 µm nylon mesh filter in cold sorter buffer (1× phosphate buffered saline, 2% newborn calf serum, 0.1% sodium azide). Single-cell preparations were resuspended in 200 µL with FcBlock. Cells were enriched as previously described [52] by staining with 10 µM 2W1S:1-Ab MHC-II tetramer, conjugated to allophycocyanin (APC), incubated at room temperature in the dark for 1 h, washed, stained with anti-APC magnetic beads (Miltenyi, Bergisch Gladback, Germany), and passed over an LS column on a quadroMACS magnet. Eluted cells were stained with the following anti-mouse antibodies: CD4 (PE), CD44 (PerCP-Cy5.5), CD8 (BV510), CD3 (fluorescein isothiocyanate [FITC]), CD11b, CD11c, CD19, and F4/80 (eFlour 450). Cells were collected on a BD FACSCelesta instrument (BD, Franklin Lakes, NJ, USA). Data were analyzed using FlowJo software (TreeStar, Ashland, OR, USA).

### 4.8. Single-Cell Suspension Generation, B-Cell Tetramer Staining, Magnetic Assisted Cell Sorting, Flow Cytometry

Spleen, mLNs, and Peyer’s patches were made into single-cell suspensions by homogenizing organs over a 100 µm nylon mesh filter in cold sorter buffer (1× phosphate buffered saline, 2% newborn calf serum, 0.1% sodium azide). Single-cell preparations were resuspended in 50 µL of FcBlock with 1 µM of PE Cy5 decoy for 5 min at room temperature, and then 1 µM of OVA-PE B-cell tetramer was added for 25 min on ice in the dark; tetramers were made using a previously established protocol [53]. Cells were washed and incubated with anti-PE magnetic beads (Miltenyi, Bergisch Gladback, Germany) and passed over an LS column on a quadroMACS magnet. Eluted cells were stained with the following anti-mouse antibodies: GL7 (eFluor 450), CD3ε, CD11c, F4/80 (BV510), IgD (FITC), CD19 (PE-Cy7), IgM (APC), and CD38 (AF700). Cells were collected on an LSRFortessa (BD, Franklin Lakes, NJ, USA). Data were analyzed using FlowJo software (TreeStar, Ashland, OR, USA).

### 4.9. Statistical Analysis

Statistical differences between data sets were assessed using GraphPad Prism software (GraphPad, San Diego, CA, USA). All data sets were tested for normality and outliers were removed using the Grubbs outlier test. Significant differences were noted as *, *p* < 0.05; **, *p* < 0.01.

## Data Availability

All data are available in the main text.
